# Supplementary material for: Content-rich biological network constructed by mining PubMed abstracts
Source: BMC Bioinformatics. 2004 Oct 8;5:147. doi: 10.1186/1471-2105-5-147 (PMC528731; doi:10.1186/1471-2105-5-147)
Supplement: Additional File 2 — The original results of the above study (non-essential files are deleted to keep the file size under the limit set by BMC bioinformatics). [file 1471-2105-5-147-S2.bz2 › chilibotAdditionalFile2/dip30/171ID9388212E586/html/IL6ST_JAK2.html]

 


 **IL6ST** and **JAK2** 
  
Found 57 abstracts in PubMed, retrieved 30.  
 

 What does Google say? 
 PDF only 
| .edu only 

---

**Interactive relationship** (e.g. stimulation, inhibition, etc)

**Neutral relationship**- Bcr gp130  [ **IL6ST** ]  is capable of interacting with JAK1,  **JAK2** , and TYK2.  Ref: 9388212 J Biol Chem, 1997
- The signal transduction of gp130  [ **IL6ST** ]  involves the Janus tyrosine kinases JAK JAK1,  **JAK2**  and Tyk2 and then the downstream effectors comprising the signal transducer and activator of transcription 3 STAT3 and mitogen activated protein kinase MAPK pathways.  Ref: 10929036 Br J Haematol, 2000
- We further show that the Janus kinases Jak1 and  **Jak2**  constitutively associated with receptor constructs containing the cytoplasmic part of LIFR, OSMR, or gp130  [ **IL6ST** ] , respectively.  Ref: 10586060 J Immunol, 1999
- Phosphorylation of  **JAK2** , STAT3, and STAT5 was observed upon the addition of lysozyme, suggesting the activation of both EpoR and gp130  [ **IL6ST** ]  derived signals.  Ref: 11481050 J Biochem Tokyo , 2001
- Importantly, tyrosine phosphorylation of  **JAK2**  is reduced in the face of increased gp130  [ **IL6ST** ]  phosphorylation, indicating impaired downstream activation of this critical pathway in DCM.  Ref: 12591746 Circulation, 2003

**Non-interactive relationship** (e.g. studied together, co-existance, homology, etc.)

- Inactivation to CNTF was not due to a decrease in CNTF receptor subunit gp130  [ **IL6ST** ]  or in levels of Jak1 or  **Jak2** .  Ref: 11882386 Cell Signal, 2002
- ITIMs with the restricted consensus sequence occur on IL 4Ralpha, IL 3Rbeta type II, gp130  [ **IL6ST** ]  cytokineR, OB R, LIF Rbeta TNF RI, G CSF R, PDGF R, Blk, Ctk Ntk, Lsk, Zap 70, PKB RACalpha, PKC alpha, PKC beta, PKC gamma, PKC delta, PKC zeta, PKC epsilon, PKC eta, PKC phi, PKC mu, calmodulin dependent kinase IIdelta, SLP 76 associated protein, FYN binding protein, Shc binding protein, RasGRF2, CDC25 homologue,  **Jak2** , Jak3, PLCbeta1, and PLCbeta3.  Ref: 10872892 Crit Rev Immunol, 2000
- Both the phosphorylation of gp130  [ **IL6ST** ]  and the association of gp130  [ **IL6ST** ]  with JAK1 and  **JAK2**  were increased after pressure overload.  Ref: 10442402 Heart Vessels, 1998
- RESULTS We showed that  **JAK2** , STATs, 5a and 6 and gp130  [ **IL6ST** ]  proteins are tyrosine phosphorylated as early as 5 30 min post MI.  Ref: 12504822 Cardiovasc Res, 2003
- Upon stimulation with IL 6 sIL 6R, the gp130  [ **IL6ST** ] , cytoplasmic Janus kinases JAK1 and  **JAK2**  were tyrosine phosphorylated.  Ref: 9610741 J Bone Miner Res, 1998
- The catalytic domain of the human  **Jak2**  was expressed and its specificity for phosphorylating peptide substrates derived from the gp130  [ **IL6ST** ] , STAT, and Jak3 molecules was determined and compared to that for human Jak1 and Jak3.  Ref: 9618263 Biochem Biophys Res Commun, 1998
- The Janus kinases Jak1,  **Jak2** , Tyk2 and gp130  [ **IL6ST** ]  show intermediate half lives.  Ref: 10491180 Eur J Biochem, 1999
- Mechanical stretch induced rapid phosphorylation of JAK1,  **JAK2** , Tyk2, STAT1, STAT3, and glycoprotein 130  [ **IL6ST** ]  as early as 2 minutes and peaked at 5 to 15 minutes.  Ref: 10347087 Circ Res, 1999
- The key components involved are the signal transducing receptor subunit gp130  [ **IL6ST** ] , the Janus kinases Jak1,  **Jak2**  and Tyk2, STAT1 and STAT3 of the family of signal transducers and activators of transcription, the protein tyrosine phosphatase SHP2 and the suppressors of cytokine signalling SOCS1, SOCS2 and SOCS3.  Ref: 10491180 Eur J Biochem, 1999
